# Differential Gene Expression in the Meristem and during Early Fruit Growth of *Pisum sativum* L. Identifies Potential Targets for Breeding

**DOI:** 10.3390/ijms18020428

**Published:** 2017-02-16

**Authors:** Annu Smitha Ninan, Anish Shah, Jiancheng Song, Paula E. Jameson

**Affiliations:** 1 School of Biological Sciences, University of Canterbury, Christchurch 8140, New Zealand; annu.ninan@canterbury.ac.nz (A.S.N.); anishmalde86@gmail.com (A.S.); jcsong88@yahoo.com (J.S.); 2 School of Life Sciences, Yantai University, Yantai 264005, China

**Keywords:** cytokinin, isopentenyl transferase, cytokinin oxidase/dehydrogenase, sucrose transporter, amino acid permease, WUSCHEL, BAM1, process pea, field pea, gene editing

## Abstract

For successful molecular breeding it is important to identify targets to the gene family level, and in the specific species of interest, in this case *Pisum sativum* L. The cytokinins have been identified as a key breeding target due to their influence on plant architecture, and on seed size and sink activity. We focused on the cytokinin biosynthetic gene family (the *IPTs*) and the gene family key to the destruction of cytokinins (the *CKXs*), as well as other gene families potentially affected by changing cytokinin levels. These included key meristem genes (*WUS* and *BAM1*) and the transporter gene families, sucrose transporters (*SUTs*) and amino acid permeases (*AAPs*). We used reverse transcription quantitative PCR (RT-qPCR) to monitor gene expression in the vegetative meristem and in pre- and post-fertilisation young pea fruits. *PsWUS* expression was specific to the shoot apical meristem while *PsBAM1* was highly expressed in the shoot apical meristem (SAM) but was also expressed at a low level in the young fruit. Differential expression was shown between genes and within gene families for *IPT*, *CKX*, *SUT*, and *AAP. PsCKX7* showed strong gene family member-specific expression in the SAM, and was also expressed in young pea fruits. We suggest that *PsCKX7* is a potential target for downregulation via molecular breeding or gene editing.

## 1. Introduction

In this International Year of the Pulses it is worth noting that *Pisum sativum* L. is one of the world’s oldest domesticated crops [1]. Eaten when mature and dried, the “field pea” has been a major source of protein for the human diet for millennia [2]. Current breeding objectives for pea vary depending on production region and end use of the crop. In New Zealand, the focus is mainly on cultivars of “process pea”, which are grown for fresh consumption for domestic use and for export as frozen baby peas. Process peas are harvested when immature and sweet. In Europe, on the other hand, the crop is predominantly “field pea” which is harvested at maturity, when starch and storage proteins have accumulated. Field peas are used for animal feed and for “mushy” peas consumed by humans.

The challenge to plant breeders is to develop crop varieties that are both more productive and more nutritious than the current varieties. Crop yield can be maximised through increased seed number and/or seed size, and quality improved potentially through the manipulation of transporters such as those loading amino acids or sugars into developing seeds [3,4,5]. The plant hormone group, the cytokinins, is strongly implicated in seed yield, both in terms of seed number and seed size [6] and early work with soybean (*Glycine max* L.) and lupin (*Lupinus angustifolius* L.) had shown that treatment with cytokinins increased pod set and/or delayed abscission of flowers and young pods [7,8,9,10,11] indicating that cytokinin might be limiting in these processes in legumes.

For example, Carlson et al. [8] showed that the application of the cytokinin 6-benzylaminopurine (6-BAP) to soybean significantly increased pod initiation by 58% and doubled the number of positions which had a 100% probability of setting pods [8]. Further, they indicated that the probability that a flower will produce a mature pod is dependent upon the total amount of cytokinin available to that flower throughout reproductive development [8]. In addition to the previous study, Dyer et al. [9] stated that there is a close association between cytokinin flux and pod set in soybean, and the ability to enhance pod set through exogenous applications of 6-BAP. They stressed that to obtain significant increases in seed yield would require either a substantial increase in pod number or the identification of mechanisms by which seed size could be maintained despite the increased reproductive load [9]. In another study, raceme tissues of soybean were treated with 6-BAP application. This caused a significant increase in flower production and seed yield over the controls [12]. However, timing of application is critical and the repeated applications that have led to yield increases in the field [13] are unlikely to be realised in cropping situations [14].

As an alternative approach, Aikens et al. [14] transformed narrow leaved lupin with isopentenyl transferase (*IPT*, the gene that codes for the key step in cytokinin biosynthesis) under a flower-specific promoter. Increased cytokinin was detected in the flowers but also in the meristem. The source of the cytokinin in the meristem was suggested to be from phloem mobile cytokinin. However, *TP12-ipt* expression was also detected in vegetative apices along with increased cytokinin. Increased branching was observed and in some cases increased pod number and yield. While the changed architecture led to increased yield in some lines, the lack of specificity of gene expression along with the translocation of cytokinin indicate that enhancing *IPT* for yield benefit may be challenging [6].

However, there have been recorded increases in yield of mutants and transgenic plants in which the endogenous cytokinin has been manipulated in the shoot apical meristem (SAM) [15,16]. The genes that code for cytokinin biosynthesis (*IPT*s) and destruction (cytokinin oxidase/dehydrogenases, *CKX*s) exist in multigene families that can be differentially expressed both spatially and temporally [6,17,18,19]. Seed number was increased in both monocots and dicots, not by increasing the expression of *IPT* but by reducing the activity of specific *CKX* gene family members in the SAM [15,16]. In rice, naturally occurring mutants of *CKX2* had increased cytokinin in the inflorescence meristem and an increased seed number and yield [15]. In *Arabidopsis*, transgenic plants with downregulated *CKX3* and *5* had significantly increased seed number due to increased flower number and silique number [16]. To target the regulation of seed number in pea requires a closer investigation of cytokinin biosynthesis and destruction in the SAM which can be achieved by monitoring the expression of *IPT* and *CKX* gene family members specifically expressing in the SAM, in the unfertilised ovule, and immediately post-fertilisation.

Research on *Arabidopsis* has revealed close interactions between cytokinins and the gene regulatory network that maintains the population of stem cells in the SAM [16,20,21]. The combined activities of the shoot apical meristem and the axillary meristems establish plant architecture, and are a key determinant of yield [22]; they share an identical organisation [22]. The gene regulatory network functioning in the meristems is called the WUSCHEL/CLAVATA feedback circuit [23]. Expression of *ARABIDOPSIS RESPONSE REGULATOR7* (*ARR7)*, *WUSCHEL (WUS)* and *CLAVATA3 (CLV3)* genes is dependent on cytokinin signalling [24]. The model developed by Bartrina et al. [16] shows a direct pathway between cytokinin blocking CLV1 (the receptor of the *CLV3* peptide [25]) leading to the release from repression of *WUS* expression. While the interaction between CLV1 and WUS is well established, recent studies showed that *BAM* (*BARELY ANY MERISTEM*) gene family members (GFMs) exhibit high sequence similarity and structure with *CLV1* [26,27]. Numchuck et al. [27] suggest that while BAMs are expressed in different domains of the SAM, they may function as redundant receptors and partially complement CLV1 function. When CLV1 is mutated, BAM receptors take over in the organizing centre, whereas they are normally negatively regulated by CLV1 [27]. Alternatively, it has been suggested that CLV1 and BAM1 function together as direct receptors in the regulatory network controlling stem cell number in the SAM [28]. Irrespective of the exact interaction, fine-tuning the WUS/CLV signalling pathway could lead to plant architecture modifications with potential benefit for crop improvement [23]. As there are very limited reports on the expression of *WUS* in pea [29] and no reports on the expression of *BAM1*, these two genes were included in this study.

In addition to enhancing seed number [15,16], it has also been shown that the levels of active cytokinins change markedly during the course of seed development, and in both monocots and dicots this appears again to be the result of the balance between the biosynthesis and the destruction of cytokinin (reviewed in [6]). Cytokinins have a pivotal role in regulating seed development, which includes promoting cell division during embryogenesis, and directing the flow and accumulation of assimilates into the seed. Studies have shown that developing seeds are a rich source of cytokinins and seeds are capable of producing their own cytokinins [6]. However, pod set and early seed development may in fact be limited by a reliance on maternally supplied cytokinin [19,30].

As the size of the seed is generally considered to be primarily associated with the initial growth of the endosperm and not with the later growth of the embryo [31,32,33], we focused on tissues at the pre-storage phase during which endosperm development, cell division, and embryo and cotyledon differentiation occur [34]. During this early phase of fruit and seed development, metabolites are required as a source of energy and for enzyme activity, so both the sucrose transporter gene family, *SUT*, and the amino acid permease gene family, *AAP*, were included in our study. Family members of both of these genes have been shown to be involved in seed development, but with research more focused on the maturation phase of seed development [4,35,36]. Both AAPs and SUTs have also been transformed into pea plants with the aim of increasing yield and/or quality [4,36,37,38,39].

Molecular breeding and gene editing require knowledge of the specific genes expressing during key stages of growth and development. As many genes exist in multigene families, knowledge of the differential expression of individual gene family members has been shown to be critical [6]. Our focus was on elucidating the expression pattern of cytokinin homeostasis genes (*IPT* and *CKX*), nutrient transporter genes (*SUT* and *AAP*) and SAM-related genes (*WUS* and *BAM1*) across different developmental stages and tissue types (temporal and spatial separation) in the meristem and during early fruit set and pod growth of pea. As *IPT*, *CKX*, *SUT*, and *AAP* are multigene families, gene expression studies were performed on multiple members of each gene family. In this work, we report the expression of *WUS* and *BAM1*, and the differential expression of the *IPT*, *CKX*, *SUT*, and *AAP* gene family members, initially identified from a pea transcriptome and then quantified using reverse transcription quantitative PCR (RT-qPCR). We identify *PsCKX7* as a potential target for molecular breeding or gene editing.

## 2. Results

**Figure 1:**
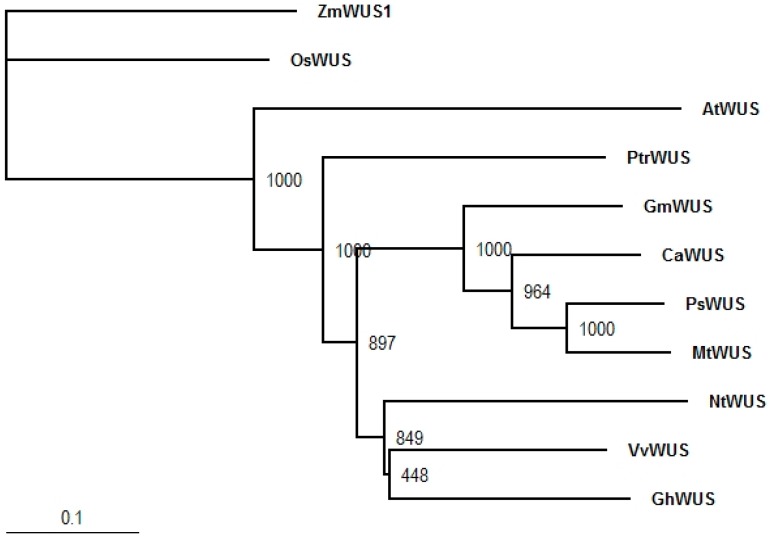
Neighbor Joining phylogenetic tree for WUS protein sequences in *Pisum sativum* L. and related species. Bootstraps values were generated with 1000 bootstrap replicates. The tree was rooted using the *Zea mays* WUS (ZmWUS1) protein sequence.

**Figure 2:**
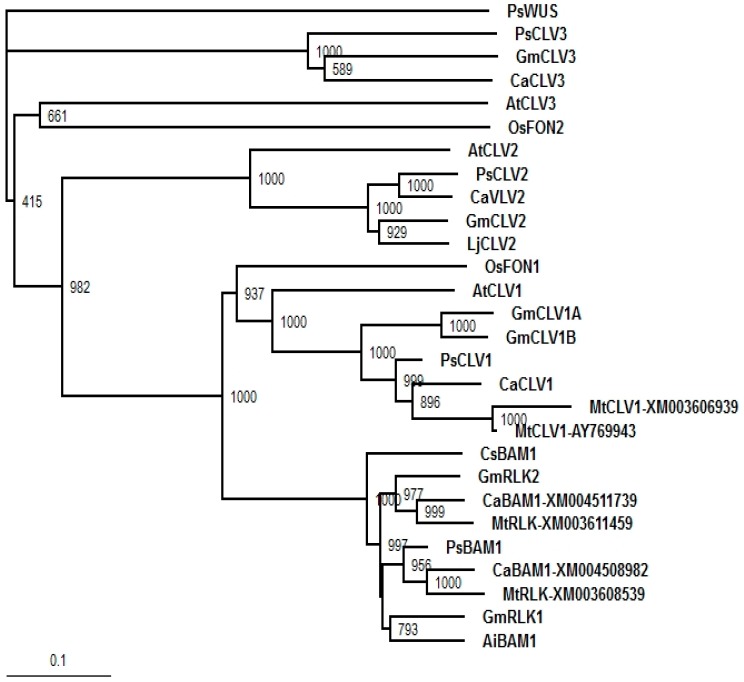
Neighbor Joining phylogenetic tree for BAM1 and CLV protein sequences in *P. sativum* L. and related species. Bootstraps values were generated with 1000 bootstrap replicates. The tree was rooted using the PsWUS protein sequence.

A pea transcriptome was mined for sequences of interest. Phylogenetic trees for *PsIPTs*, *PsCKXs*, *PsSUTs*, and *PsAAP1* are shown in Dhandapani et al. [40]. The phylogenetic trees for *PsWUS* and *PsBAM1* are shown in Figure 1 and Figure 2. One *WUS* sequence was identified which aligned with other *WUS* genes from legumes (Figure 1). One *BAM1* sequence was identified which aligned closely with other legume *BAM1* sequences sharing high sequence similarity to *CLV1* genes (Figure 2).

RT-qPCR expression data for the process pea (Bolero) is presented in detail below. Data for the field pea (Bohatyr) is presented in Figure S1. Although these are very distinctive cultivars, selected for different end purposes, they show a high degree of similarity in the expression of the targeted gene family members at their early stages of development (Figure S1). However, some differences are apparent. For example, for both *PsIPT1* and *2*, expression was slightly greater in the pod walls of the field pea (Bohatyr) than the process pea (Bolero) and *PsCKX* expression was greater in the early fruit stages of Bolero relative to Bohatyr (Figure S1). These differences may be cultivar specific, or be due to the cultivars not being harvested at exactly identical stages of development during this early rapid phase of development.

### 2.1. Differential Gene Expression in Fruits

**Figure 3:**
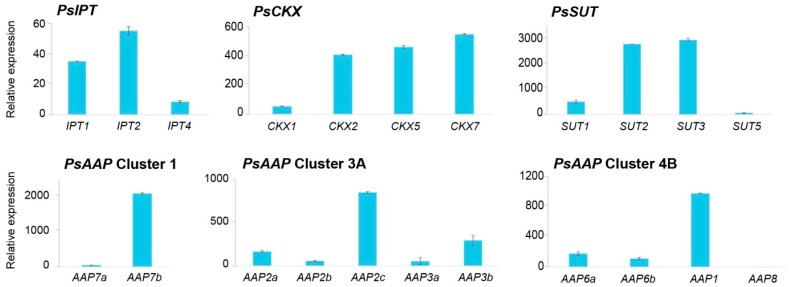
Relative expression of gene families in the ovule of *P. sativum* L. cv Bolero plants one day before fertilisation. Data are from reverse transcription quantitative PCR (RT-qPCR) and are given as fold-change values relative to the reference genes *PsEF*, *PsGAP*, and *PsACT*. *IPT*: isopentenyl transferase; *CKX*: cytokinin oxidase/dehydrogenase; *SUT*: sucrose transporter; *AAP*: amino acid permease. The results are expressed as ± standard deviation (SD).

**Figure 4:**
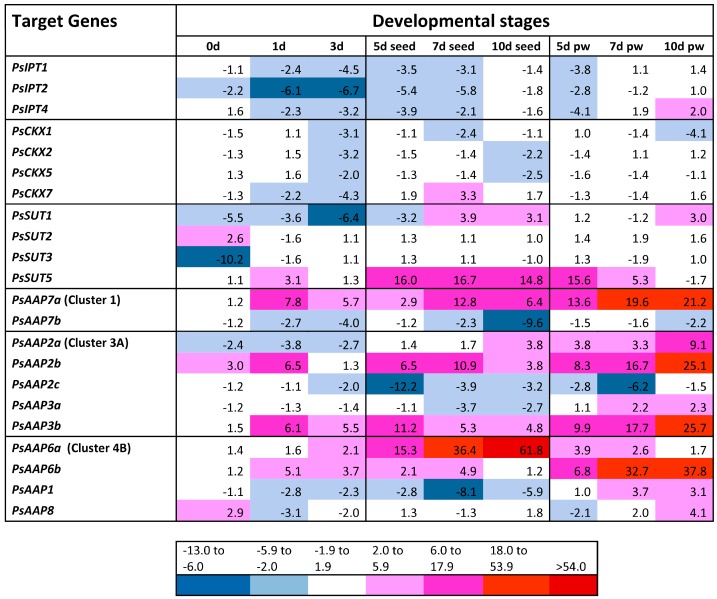
Relative expression of gene family members in developing fruits of *P. sativum* L. cv Bolero. 0 day, 1 day, and 3 day: fruit at 0, 1 and 3 days after fertilisation (DAF); 5 day, 7 day, and 10 day seed: cotyledon plus seed coat; 5 day, 7 day, 10 day pw: pod walls separated from the seed. The colour scale indicates upregulated expression (**red** scale), similar (**white**) and downregulated expression (**blue** scale) relative to one day before fertilisation.

Two of the three *PsIPT* gene family members were expressed more strongly in the unfertilised ovules of pea flowers (Figure 3). Relative to the day before fertilisation (−1 day after fertilisation, DAF), expression of *PsIPT2* decreased in the fruits immediately after fertilisation, but increased to a similar pre-fertilisation level in the pod walls 7 to 10 DAF (Figure 4). Expression of *PsIPT4* was noticeable in pod walls by 10 DAF. Of the four *PsCKX* gene family members detected, *PsCKX2*, *5* and *7* were expressing in the ovule at −1 DAF (Figure 3). Expression was then relatively consistent with respect to −1 DAF, but with *PsCKX7* showing greater expression in the seeds compared to the young pods (Figure 4).

Of the four sucrose transporters, *PsSUT2* and *3* were strongly expressed at −1 DAF (Figure 3). *PsSUT3* showed strong expression over time with the exception of sharply decreased expression at 0 DAF; a similar but less strong pattern was seen for *PsSUT1*. *PsSUT5* showed strongly increased expression in both the developing fruit and pod walls after fertilisation, and particularly in the seed. *PsSUT2* was more-or-less constitutively expressed in all tissues (Figure 3 and Figure 4).

Strong differential expression is shown amongst the 11 *PsAAP* GFMs, and even within clusters. *PsAAP* GFMs were markedly differentially expressed at one day before fertilisation, with Cluster 1 *PsAAP7b*, Cluster 3A *PsAAP2c* and Cluster 4B *PsAAP1* strongly expressed, and most of the other 11 GFMs expressed weakly (Figure 3). Relative to −1 DAF, Cluster 1 *PsAAP7a* was strongly expressed in all fruit tissues, and particularly so in the pod walls, where *PsAAP7b* showed somewhat reduced expression. Cluster 3A gene family members *2b* and *3b* were expressed in all tissues, but again most strongly in the pod walls, whereas, relative to −1 DAF, *PsAAP2c*, and *7b* generally showed reduced expression (Figure 4). Comparatively, only *PsAAP6a* was more strongly expressed in the developing seeds relative to other tissues (Figure 4). *PsAAP8* showed constitutively low expression in all samples (Figure 3 and Figure 4), until 10 DAF in pod walls.

### 2.2. Gene Expression in the Shoot Apical Meristem

**Figure 5:**
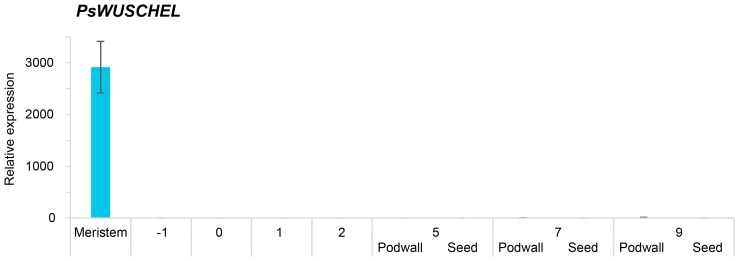

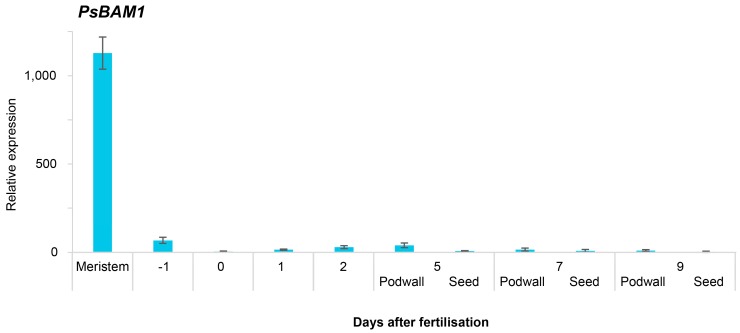
Expression of *WUSCHEL* and *BAM1* in tissue enriched with shoot apical meristems of *P. sativum* L. cv Bolero. Samples at −1, 0, 1 and 2 DAF included the entire fruit; Samples at 5, 7 and 9 DAF included pod walls separated from the seeds. The seed sample included cotyledon plus seed coat. Data are from RT-qPCR and are given as fold-change relative to reference genes *PsEF*, *PsGAP*, and *PsACT*. The results are expressed as ±SD.

Shoot tip material was dissected from young shoots of Bolero and Bohatyr to provide a source of tissue enriched in shoot apical meristems. Both *WUS* and *BAM1* were highly expressed in the meristematic tissue of Bolero (Figure 5) and Bohatyr (data not shown).

**Figure 6:**
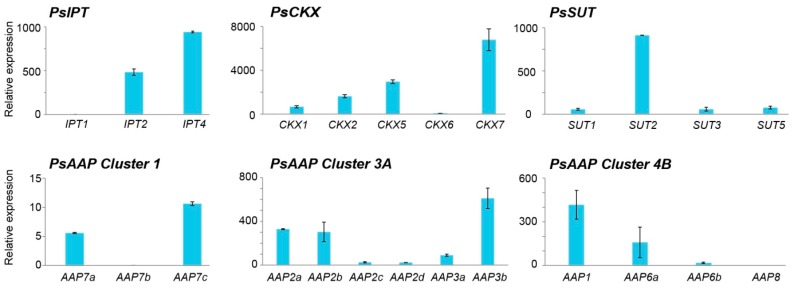
Relative expression of gene families in the shoot apical meristem of *P. sativum* L. cv Bolero. Data are from RT-qPCR and are given as fold-change values relative to the reference genes *PsEF*, *PsGAP* and *PsACT*. The results are expressed as ±SD.

*WUS* expression was specific to the meristematic tissue, while *BAM1* expression was detected in ovule, pod wall, and seed but at some 100-fold less compared to enriched meristem samples (Figure 5). *PsIPT2* and *4* were expressed in the meristematic tissues, as were elevated expression levels of *PsCKX7*, *5* and *2*, lesser levels of *CKX1*, but much reduced levels of *CKX6* (Figure 6). Strong expression of *PsSUT2* was observed in the meristematic tissue, but relatively much lower expression of *PsSUT1*, *3* and *5.* Relative to seeds and pod walls, there was low expression of the *PsAAP* gene family members in the young meristematic tissue, although expression was detected for *PsAAP 2a*, *2b*, *3b*, *1*, and *6a* (Figure 6).

## 3. Discussion

In agreement with models depicting interactions between *IPT*, *CKX*, *WUS*, and *CLV1* [16], specific *IPT* and *CKX* gene family members expressed concurrently with *WUS* and *BAM1* in the vegetative SAM of pea. As reported for *Arabidopsis* (e.g., [41]) and soybean [42,43], expression of *WUS* was specific to the SAM. However, *BAM1* showed low level but consistent expression as pods and seeds developed which aligns with the non-specific expression shown for *CLV1* and *BAM1* in *Arabidopsis* [26]. As the regulation of meristem size is governed by a conserved mechanistic framework [22], it is highly likely that this is operating in pea, and as such can be the target for manipulation of plant architecture.

During seed development the dominant sinks for nutrient loading shift from maternal tissues (the pod walls and seed coats) early in seed development to filial tissues (e.g., the cotyledon in pea) later in development [35]. At the pre-storage stage of pod and seed development, the transported sucrose is likely to be used as a source of energy but also, once inverted to glucose and fructose, as a source of osmoticum [44,45]. The transported sugar is also strongly implicated in cell division along with cell wall-bound invertase (CWINV) and cytokinin [6]. While *PsSUT2* and *3* were more-or-less constitutively expressed pre-and post-fertilisation, potentially supplying sucrose for energy metabolism, both *PsSUT1* and *5* were upregulated in the seed post-fertilisation and may be supplying sucrose for osmotic purposes and/or cell division. During the pre-storage phase the elongating pods and developing seeds are competing for metabolites. Consequently, upregulation of specific *SUT* family members (such as *PsSUT5*) may enhance the competitive capacity of the seed.

While the pea amino acid permeases have been allocated to phylogenetic clusters (see [40]), the expression of the *PsAAPs* during early seed growth was not restricted to any particular gene cluster. Some tissue specificity was noted, with the gene family members most strongly expressed in unfertilised ovules showing reduced expression post-fertilisation. Expression of others increased post-fertilisation with expression generally more strongly associated with pod walls rather than seeds, with the exception of *PsAAP6a*, which was strongly expressed in seeds.

Interestingly, AAP8 in *Arabidopsis* was recently described by Santiago and Tegeder (2016) as “the long sought after phloem loader” [46]. *AtAAP8* was shown to be expressed in *Arabidopsis* during early embryo development [47] and, more recently, in the phloem of sink and source leaves during both vegetative and reproductive growth, and in siliques [46]. However, *PsAAP8* was barely expressed during the maternal phase of pod and early seed development (Figure S1), and not detected in the vegetative SAM of pea, or in shoots of germinating seeds [48], whereas other family members from the same cluster are strongly upregulated in the unfertilised ovule (*PsAAP1* more than 800-fold relative to *PsAAP8*), the developing seed (*PsAAP6a*), and pod wall (*PsAAP6b*), and are present in the vegetative SAM (*PsAAP1* and *6a*), and in germinating seeds [48]. As with *IPT4*, which is expressed in *Arabidopsis* but not in *Brassica* species [19,49,50,51], it is clear that genes in each individual species need to be identified to family level prior to targeting for breeding purposes.

The relatively limited number of *IPT* gene family members expressed in meristems and developing pods of pea is in agreement with similarly few *IPT* gene family members expressing in pods and seeds of soybean. [52]. This was the case in both the process pea (Bolero) and the field pea (Bohatyr) (Figure S1). The low *IPT* expression post-fertilisation strongly supports previous data from lupin [30] and *Brassica napus* [19] that early development of the pea fruit is dependent on a maternal supply of cytokinin. It also indirectly supports the application experiments where applied cytokinin led to increased pod set [7,8,9,10,11,12,13].

The expression of the *CKX* gene family members is interesting. It is generally accepted that where cytokinin levels are elevated or there is increased expression of *IPT*, that *CKX* will also be expressed [19,48,53,54]. *PsCKX2*, *5*, and *7* were strongly expressed at 1 day before fertilisation (when *IPT* expression was noted) but also at 1 DAF when *IPT* expression was reduced. We interpret this activity as indicative of the presence of cytokinin translocated from maternal tissues to the developing fruit, with CKX acting to modify incoming cytokinin levels, as suggested also occurs in *B. napus* [19].

There are now several reports that suggest that CKX acts to limit seed development [18,51] or has a controlling influence on seed yield [16], with specific *CKX* gene family members identified as potential targets for breeding in rice [15], wheat [54], barley [55], and *Brassica napus* [19]. Bartrina et al. state “the role of *CKX* genes in determining yield has been evolutionarily conserved and is of functional significance for all or most flowering plants” [16]. In the case of pea, we highlight the gene family member-specific expression in the SAM of *PsCKX7*, and its expression in the fruit pre- and post-fertilisation. Interrogation of non-GE TILLING populations for mutations in *PsCKX7*, or utilisation of genome editing tools such as CRISPR/Cas9, targeted to the deletion of *PsCKX7*, may result in the required elevation of endogenous cytokinin and increased pod number indirectly via an effect on the SAM and plant architecture. More directly, an increase in fruit set and an enhanced sink size and potentially sink strength may also result. Coordinated increase in expression of appropriate GFMs of both *SUT* and *AAP* transporters, as seen in Figure 4, will need to occur to maintain seed number and seed size.

In conclusion, we have clearly identified the differential activity of genes and gene family members during the pre-storage stage of pea development, and identified genes and/or gene family members that could be the targets for molecular breeding. However, in targeting such genes care must be exercised to determine that off-target effects have not occurred, such as, in the case of increased cytokinins, decreased root growth [17], delayed senescence (which may impact final yield positively or negatively [6]), or an impact on germination and seedling growth [48].

## 4. Materials and Methods

Seeds of *P. sativum* L. cv. Bolero (a “process pea”) and Bohatyr (a “field pea”) were sourced from The New Zealand Institute for Plant & Food Research. Both cultivars represent the “industry standard” and are pure lines. The seeds were planted in 2.5 L pots (one seed per pot) filled with a commercial potting mix with slow release fertiliser. The plants were maintained in an unheated glasshouse, with a temperature range from 15 to 22 °C. Plants were sown in early autumn (March, Christchurch, New Zealand) under the prevailing photoperiod.

After several weeks’ growth, each plant had a number of flowers and pods. These were date-labelled using paper strips, according to when the flowers opened, which indicated the time of fertilisation. The petals of a flower just opened were marked as 0 DAF. Flower labelling was done in such a way as to ensure collection of sufficient samples ranging from very young (unopened) flowers through to fully developed pods. More specifically, the developmental stages which were used for the gene expression studies were as follows: −1/−2, 0, 1, 2, 5, 7, and 9/10 DAF. Petals were removed from the samples, so that only the tiny ovules or seeds and seed pods were extracted. The seeds were able to be separated from the pod walls at stages 5, 7, and 9 DAF. All samples were frozen in liquid nitrogen as soon as they were removed from the plant and stored at −80 °C prior to RNA isolation. This method of labelling and sampling was consistent across both pea cultivars.

Seeds of Bolero and Bohatyr were also grown in petri dishes in the lab. This was done to obtain very young shoots from which the shoot tip could be dissected. The dissected shoot tips served as an enriched tissue for the SAM.

Target gene sequence identification was based on an RNA-Seq transcriptomic data set as described in Dhandapani et al. [40]. Sequences of candidate gene family members were isolated from RNA-Seq transcriptome data. A pool of total RNA samples extracted from multiple tissue including shoot apical meristems, leaves, flowers, and pods was used to construct the cDNA library, which was then sequenced using an Illumina HiSeq 2000 genome analyser at the Beijing Genomic Institute (Shenzhen, China) customer service.

Orthologue sequences of *WUS*, *BAM1*, *IPT*, *CKX*, *SUT*, *AAP*, and *CWINV* from *Arabidopsis* and leguminous species available in the GenBank database were used as query sequences to BLAST search our field pea cv Bohatyr transcriptome data set using prfectBLAST 2.0. The putative sequences were verified via BLAST searching the GenBank database and multiple sequence alignment with representative orthologue sequences in closely related species. The newly identified sequences and their orthologues in leguminous species were used to construct Neighbor Joining (NJ) phylogenetic trees using ClustalX software with 1000 bootstrap replicates. Each tree was rooted with an out group orthologue sequence. The GenBank accession number for the nucleotide sequence for *PsWUS* is BankIt1974292 PsWUS KY312112 and for *PsBAM1* it is BankIt1974292 PsBAM1 KY312113. Other accession numbers are listed in [40].

RNA isolation, cDNA synthesis and real-time reverse transcription quantitative PCR (RT-qPCR) were as described in Dhandapani et al. [40]. Data are the averages of three technical replicates, with the data for Bolero and Bohatyr providing the biological replication. The relative expression (fold change) of each target gene was corrected using the geometric mean of the two reference genes, *PsELONGATION FACTOR* (*eEF-1α*) and *PsGAPDH*, and calculated using the 2^−∆∆^*C*_t_ method as described in [18,19]. For ease of comparison, much of the expression data is presented as a heat map with values calculated in fold-change relative to one day before fertilisation (−1 DAF).

## Supplementary Materials

Supplementary materials can be found at www.mdpi.com/1422-0067/18/2/428/s1.
