# Supplementary material for: Differential Gene Expression in the Meristem and during Early Fruit Growth of Pisum sativum L. Identifies Potential Targets for Breeding
Source: Int J Mol Sci. 2017 Feb 16;18(2):428. doi: 10.3390/ijms18020428 (PMC5343962; doi:10.3390/ijms18020428)
Supplement: Supplementary file 1 [file ijms-18-00428-s001.pdf]

Supplementary Materials: Differential Gene Expression in the Meristem and during Early Fruit Growth of *Pisum sativum* L. Identifies Potential Targets for Breeding

Annu Smitha Ninan, Anish Shah, Jiancheng Song and Paula E. Jameson

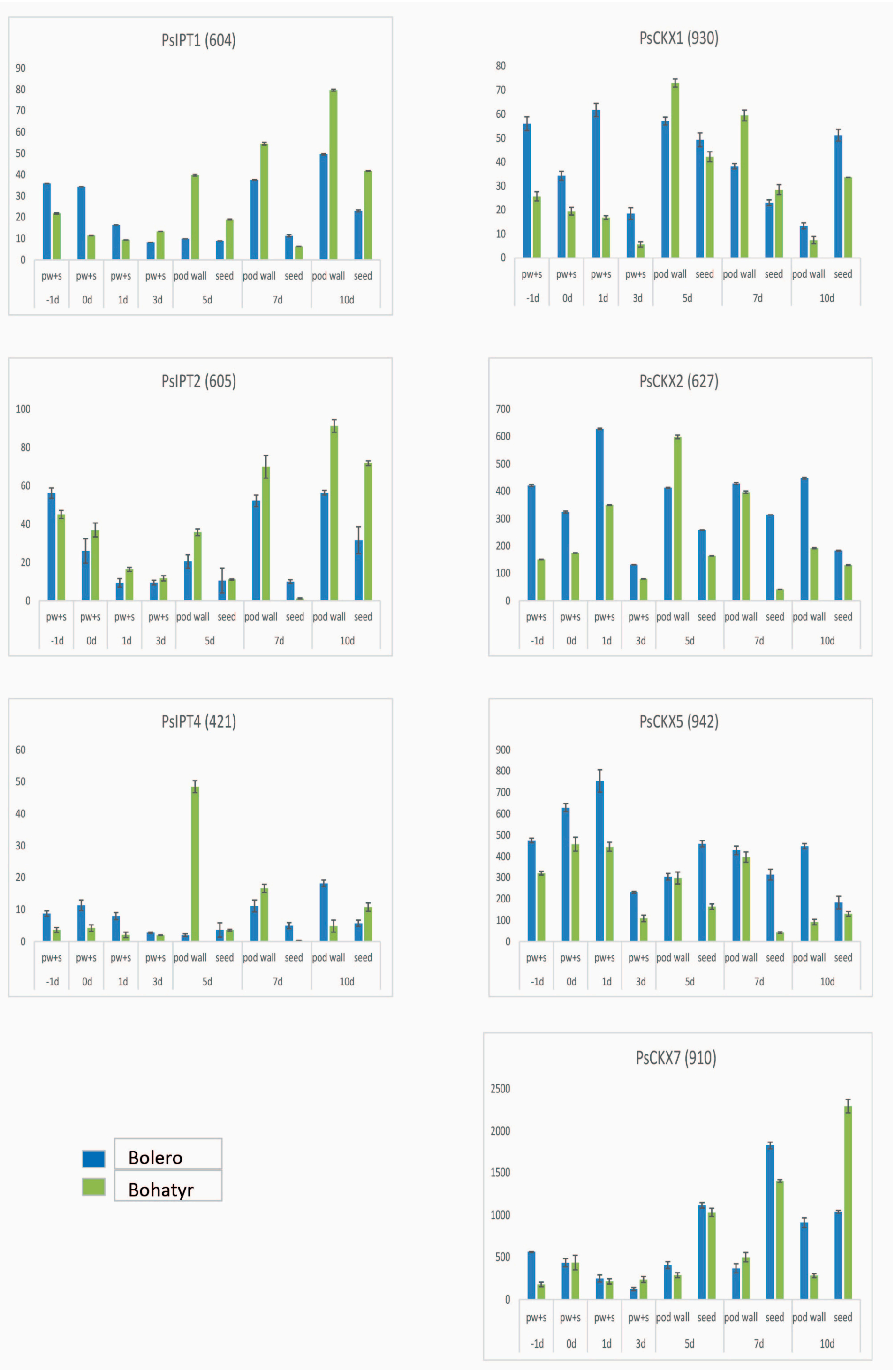

Figure S1. *Cont.*

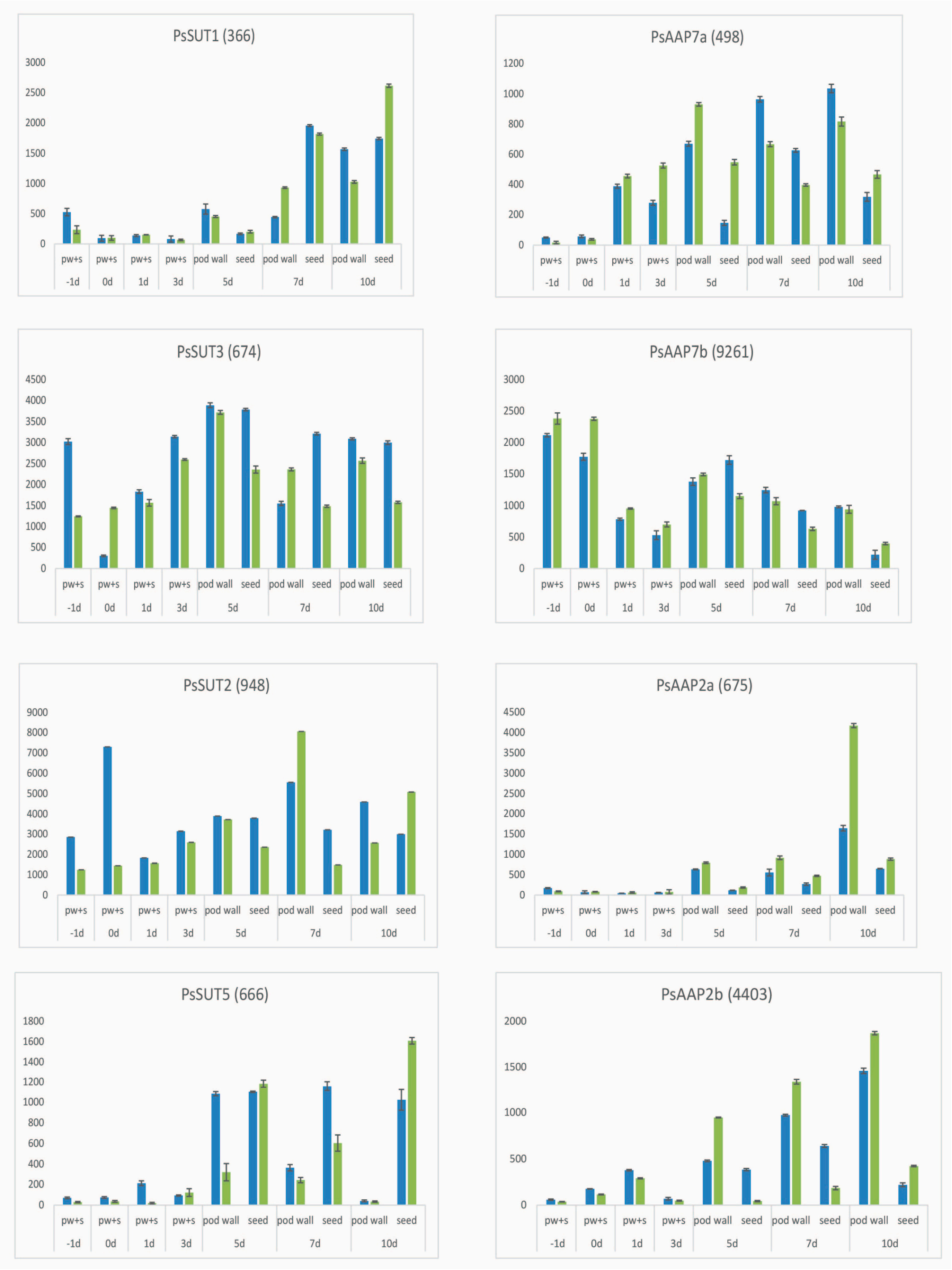

Figure S1. *Cont.*

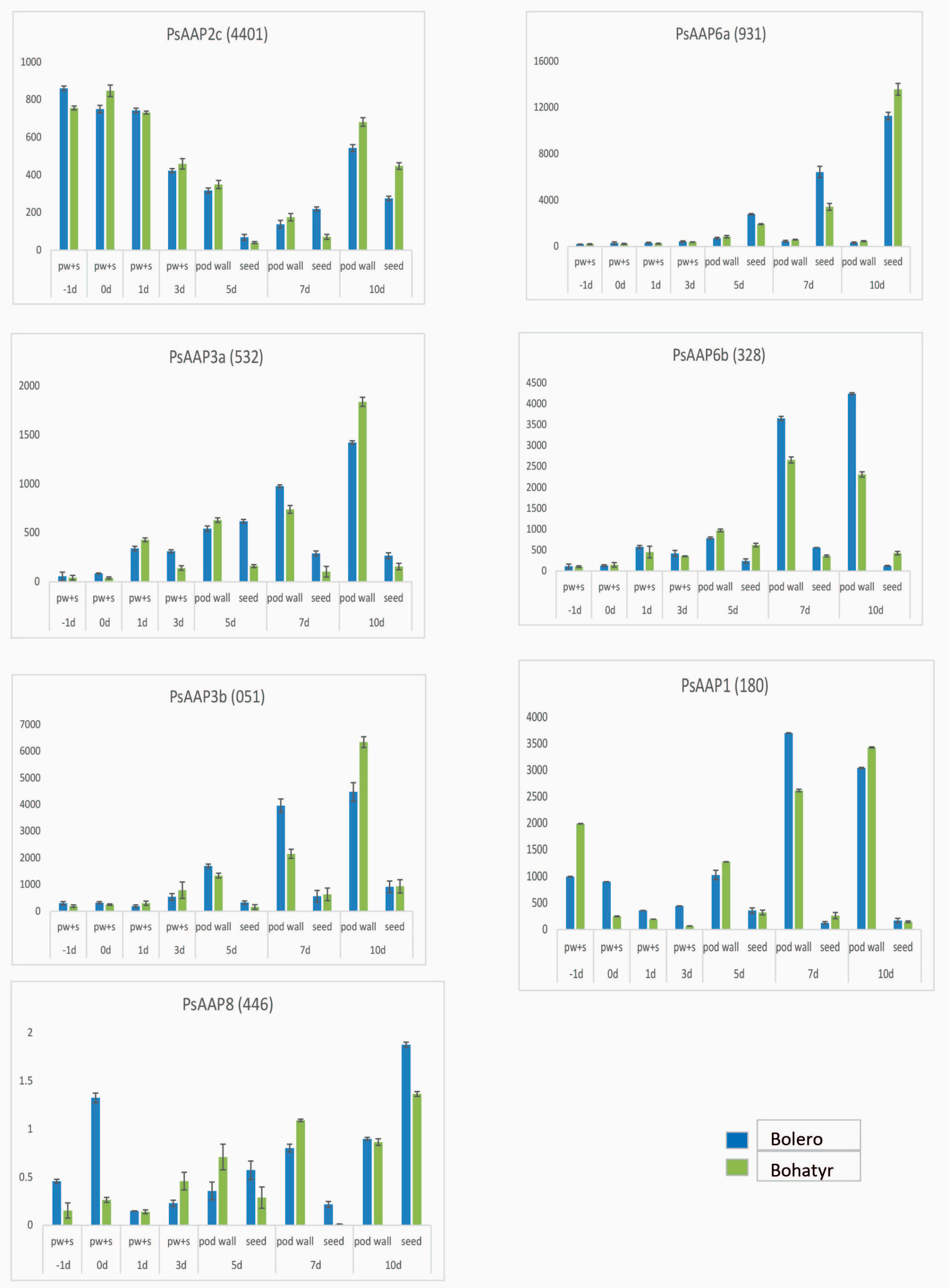

Figure S1. RT-qPCR expression of *PsIPTs*, *PsCKXs*, *PsSUTs* and *PsAAPs* in *Pisum sativum* L. cv Bohatyr and Bolero.
